# Palliative care: Progress, needs, and challenges

**DOI:** 10.1186/2045-4015-1-10

**Published:** 2012-02-20

**Authors:** Barrie R Cassileth

**Affiliations:** 1 Integrative Medicine Service, Memorial Sloan-Kettering Cancer Center, 1429 First Avenue, New York, NY 10021, USA

## Abstract

Palliative care is increasingly available and the importance of its role increasingly recognized. International work toward making palliative care a basic human right underscores the growing need to ensure comfort and pain relief for the terminally ill. The organizational structures in place for providing such care vary greatly within and across countries; even definition of the term is not uniform. The World Health Organization (WHO) definition includes the statement that palliative care "... intends neither to hasten nor postpone death...", thus illustrating varying socio-cultural perceptions. In addition to cultural differences, other challenges include clinical, economic, and varying institutionalized systems and practices in patient care.

This is a commentary on http://www.ijhpr.org/content/1/1/9/

## Commentary

The important paper by Bentur et al. [1] captures the range of challenges to the inclusion of palliative care in medicine. Various clinical, economic, cultural, and institutionalized systems and practices all impinge on the ability of Israel and other countries to bring about this needed component. Moreover, the effort to accomplish that goal occurs in the context of simultaneous efforts by other groups to deal with the same problems that are targeted by the Palliative Care medical subspecialty.

This brief Commentary touches on: (1) palliative care terminology and its extent world-wide, (2) ethical and human rights perspectives, and (3) potential collaborations and other means of reaching palliative care goals.

## Palliative care terminology: varying international perspectives

The term "palliative care" literally means palliation or comfort provided to the ill. The article to which this Commentary responds gives the definition as "... palliation for anyone who needs it, no matter their prognosis." However, despite this statement and effort in the Palliative Care community to extend its purview to patients at all stages of illness, the internationally common use of the term refers to the relief of physical and emotional suffering among the terminally ill. Managing pain and other physical or emotional distress in patients under active treatment geared to remission or cure is commonly termed "symptom management" or "supportive care", rather than "palliative care". While it may seem reasonable to combine the two, doing so may lead to less than optimal results as the unique requirements of each may be left unmet.

The World Health Organization (WHO) defines palliative care as the provision of "... relief from pain and other distressing symptoms that affirms life and regards dying as a normal process; (and) intends neither to hasten or postpone death..." [2].

This definition of terms in the WHO document exemplifies the international focus on palliative care. Through numerous national and regional groups, such as the European Association of Palliative Care, comfort for the terminally ill has become a truly international effort. This is evident also in the fact of many international organizations that strive to insure comfort for those facing end-of-life circumstances. Examples include:

• Cairdeas International Palliative Care Trust

• Children's Hospice International

• International AIDS Society (IAS)

• International Association for Hospice and Palliative Care (IAHPC)

• International Association for the Study of Pain (IASP)

• International Brain Tumor Alliance (IBTA)

• International Children's Palliative Care Network (ICPCN)

• International Observatory in End of Life Care (IOEL)

• International Palliative Care Family Carer Research Collaboration (IPCFRC)

• International Palliative Care Initiative - Open Society Institute

• Pain and Policy Studies Group - WHO Collaborating Center

• Palliativedrugs.com Ltd

• The International Network for Cancer Treatment and Research (INCTR)

• World Institute of Pain (WIP)

• Worldwide Palliative Care Alliance (WPCA)

## Palliative care from an ethical and human rights perspective

The international palliative care community has moved to make palliative care a basic human right [3]. A world-wide map of palliative care activity, developed by the International Observatory on End-of-Life Care for the Worldwide Palliative Care Alliance, documents varying levels of effort and success [4]. The International Covenant on Economic, Social and Cultural Rights, a component of the International Bill of Rights, includes palliative care and pain management as "core obligations" [3]. The addition of a moral or ethical obligation is especially important. It may help counteract cultural traditions requiring that all patients receive aggressive and often painful treatments that have no possibility of restoring health. The essence of palliative care is the relief of pain and suffering.

Although palliative care has been deemed an essential public health component, mandating its availability has proven problematic. For example, in early 2011, a New York State law added a "Palliative Care Information Act" to its Public Health Law. It requires physicians and nurse practitioners to offer terminally-ill patients information and counseling about palliative care and end-of-life options. Palliative care, as defined by the law, is "health care treatment... and consultation with patients and family members, to prevent or relieve pain and suffering and to enhance the patient's quality of life, including hospice care." [5] California passed a similar law two years earlier, and other U.S. states plan to follow suit. Although the aims of such laws are laudatory, there are major obstacles to their implementation.

Not all practitioners possess the compassion, communication skills, and knowledge base necessary for achieving the desired consultative processes. Moreover, many physicians see legal requirements as an unwelcome and unhelpful intrusion into the doctor-patient relationship. Finally, many patients do not want to shift caregivers from their long-term physicians to newcomers, and transitions to palliative care experts is often perceived as abandonment by their existing, trusted medical professionals.

Although the goals are important, the widespread provision of palliative care awaits a cultural shift toward acceptance and improved skills among medical professionals as well as patients and families. Hope is not only for living longer. Patients also hope for comfort, for realistic information, for the opportunity to take care of family matters, and for caring and compassion.

## Collaborations and other means of reaching palliative care goals

When William Jefferson Clinton was president of the United States, he dealt with at least two very different Middle East problems: political turmoil in the region and inequities in the treatment of cancer in Israel compared with its Arab neighbor states. With respect to the latter, he formed the Middle East Cancer Consortium (MECC) in Israel to spread knowledge about optimal cancer treatment, including palliative care, and by so doing, perhaps further cooperation in the region [6].

Annually since 2004, MECC's Palliative Care conferences have enabled health professionals from throughout the Middle East to work together toward better end-of-life care. With sensitivity to both cultural differences and structural or organizational variations, MECC has managed to contribute to the reduction of tensions and the development of collegial relationships, achieving at least to some degree its dual aim to improve both the political and the health care situations. MECC members include the Health Ministries of Cyprus, Egypt, Israel, Jordan, Palestinian Authority, and Turkey. MECC is an example of successful efforts to bridge the cultural, religious, and health-care gap in promoting palliative care goals.

Formal palliative care groups offer substantial help to terminally ill patients. Other groups also contribute to the effort. Some do so under the umbrella of supportive care; others, within broad integrative medicine programs such as ours at Memorial Sloan-Kettering Cancer Center, employ non-pharmacologic means to reduce physical and emotional suffering in collaboration with the patient's treating physician. That physician also may prescribe medications if required.

There appear to be multiple routes to the provision of helpful palliative care. All require compassion and sensitivity to inter-patient differences, plus the ability to communicate appropriately with each individual and his family. All require sensitivity to local belief systems and traditions. These are difficult but not impossible goals. The future of palliative care internationally is promising.

## Competing interests

The author declares that she has no competing interests.

## Author's information

Barrie R Cassileth founded the Integrative Medicine Service at the Memorial Sloan-Kettering Cancer Center, where she remains the chief, and holds the Laurance S. Rockefeller Chair in Integrative Medicine.
